# Training Adolescents and Young Adults to be Partners in Research: Co‐Creating the Young Patients' Autoimmune Research and Empowerment Alliance

**DOI:** 10.1111/hex.70642

**Published:** 2026-04-05

**Authors:** Anya R. Khurana, Kaye Anderson, Kristine Carandang, Ela Chintagunta, Jennifer Ziegler, Courtney K. Wells

**Affiliations:** ^1^ Young Patients' Autoimmune Research and Empowerment Alliance Young Patients' Autoimmune Research & Empowerment Alliance San Diego California USA; ^2^ Social Work Department University of Wisconsin‐River Falls River Falls Wisconsin USA

**Keywords:** adolescent, autoimmune disease, patient engagement, patient‐centred, research design, research involvement, young adult

## Abstract

**Background:**

Adolescents and young adults (AYAs) are frequently excluded from efforts to include patient research partners due to systemic and procedural barriers and lack of awareness by both researchers and patients. For meaningful collaboration to occur, patient partners must be prepared with appropriate knowledge, skills, and confidence.

**Objective:**

To co‐design a model that trains AYAs with autoimmune conditions to be research partners and to evaluate feasibility for future application in other settings.

**Methods:**

Together with the Project Team, 12 AYAs with autoimmune conditions (16–22 years old) co‐created the Young Patients' Autoimmune Research and Empowerment Alliance (YP AREA), a council aimed at elevating young patients' voices in research. AYA Council Members (CMs) completed two stages of training: (1) Educate (exposure to the research process) and (2) Empower (practice with advocating) and received 1–1 mentoring focused on individual goals. Two surveys (the Patient Engagement in Research Scale and the Research and Empowerment Survey) and qualitative interviews were conducted at the end of each stage to assess research readiness, empowerment, and perceptions of meaningful collaboration.

**Results:**

After training activities, CMs reported personal growth and feeling like they were ready to partner with researchers. CMs highlighted the importance of community with like‐minded peers. Survey scores showed that CMs felt highly engaged in the group process and had high levels of psychological empowerment and research readiness after trainings.

**Discussion:**

Young patients can obtain high levels of engagement in the research process. This intentional training process was designed to meet AYAs at their developmental stage through individual mentoring, hands‐on group activities, and community building. As a result, AYAs were empowered to advocate within research and academia for the healthcare needs of their communities.

**Patient or Public Contribution:**

All Project Team Members and Council Members identify as patients with autoimmune conditions. Young patients co‐designed all the activities included in this manuscript unless otherwise stated, co‐conducted the evaluation, and co‐authored this manuscript.

**Abbreviations**
AYA: adolescent and young adult
CM: council member
YP AREA: Young Patients' Autoimmune Research and Empowerment Alliance

## 1 Introduction

Authentic engagement of patient partners has the potential to improve how health research is conducted, thereby advancing scientific knowledge and the ability to target health outcomes [1]. In this manuscript, we use the term “*patient partner*” to describe a person who contributes their expertise with a health condition and as a consumer in a healthcare system to advance the planning and execution of a research study as a member of the research team. While there is a growing amount of literature surrounding patient engagement models [2], there is no comprehensive guide for how to engage young people in health research [3]. Within this paper, we present our method for co‐creating Young Patients' Autoimmune Research and Empowerment Alliance (YP AREA), a council aimed at training adolescents and young adults (AYAs; defined for this project as 16–22 years old) with autoimmune diseases to be patient partners for a variety of research projects. In doing so, we hope to provide others with a starting point for how to achieve meaningful collaboration with AYAs in the development of research projects, healthcare programming, and health policy.

AYAs have the lowest healthcare utilisation (with the exception of high emergency department visits) compared to other age groups as well as low knowledge about healthcare navigation, insurance, and their personal rights [4, 5, 6, 7]. These gaps cause significant challenges for AYAs with chronic conditions who require a smooth transfer from paediatric to adult specialty care to maintain health and medication management. For over three decades, researchers and healthcare providers have tested interventions, clinic processes, and policies to support AYAs [8, 9, 10, 11]; however, both young patient outcomes and the effectiveness of related healthcare in the United States remain poor [4, 12, 13, 14, 15, 16, 17, 18]. To interrupt this cycle, systems must be restructured to meet the needs of AYAs. Patient‐engaged research presents an avenue to achieve this structural change by empowering AYAs to participate, contribute, collaborate, and lead research that is for and about them [19, 20, 21].

It is widely advocated that AYAs be included as research collaborators as both an ethical issue and to increase the relevance and effectiveness of research programmes [22, 23, 24, 25, 26]. AYAs are primed to contribute to research processes; their emergent cognitive and social skills demand creativity, passion, and meaningful engagement [20]. They are coming of age in a dramatically different environment than previous generations and have unique ways of communicating and managing their health. AYA patient partners with chronic health conditions offer insight into how young people interact with healthcare professionals, the burdens that they face in doing so, and possible solutions to these challenges, all of which may serve as a starting point to study design and outcome selection [23]. Patient engagement may also be an avenue to fulfil AYAs' developmental needs [27, 28, 29]. Benefits include enhancing communication, leadership, and critical thinking skills; promoting autonomy and identity development; connecting with peers and adult role models; and encouraging civic engagement [27, 30, 31, 32]. Participation in research may help AYAs move from feeling powerless over their health conditions towards a state of empowerment.

Despite these benefits, a recent scoping review found that less than 1% of youth‐focused studies included the input of young people in their study design and execution [21]. There are several possible reasons for the lack of ideal youth engagement. First, most research findings and discussions are inaccessible to patient communities due to paywalls, lack of targeted communication, and research jargon, thereby already excluding patients from understanding and engaging with research. Second, successful collaborations between researchers and patient partners require intentional methods to train the entire team and build capacity for patients' expertise to be fully realised, but academic environments offer minimal incentives for researchers to use patient‐engaged methods [26, 33, 34]. Patients must feel adequately informed and empowered to successfully fulfil their role as a patient partner [34, 35, 36], and young patients may require additional support due to limited experiences with work of this nature [37]. Researchers must be knowledgeable on how to intentionally plan their methods of patient engagement to avoid tokenism and marginalisation [38, 39, 40, 41], especially as unequal power dynamics are inherent in the difference in age and educational attainment between AYAs and other members of the research team. While dedicating time and personnel to these efforts have led to successful collaborations, researchers may not be afforded these resources within their funding and career timeline. Finally, researchers may face institutional barriers such as issues of privacy, lack of understanding from Institutional Review Boards, the need for informed consent, and when working with minors, parent permission [20, 27]. These obstacles deter researchers from engaging AYAs in meaningful ways, ultimately disempowering patients in their own health management and hindering the progress of AYA‐centred care.

To combat these problems, YP AREA empowers its AYA Council Members (CMs) through didactic and experience‐based learning to become research‐ready collaborators who are trained to advocate for themselves and their communities in research settings. Contrary to many groups, YP AREA is not affiliated with one specific research study, agenda, or setting. In our model, any health researcher may request to collaborate with YP AREA as a whole or to work with individual CMs who fit their needs for expertise. CMs may also identify and pursue their own research avenues with the support of PhD‐level project team members.

The development of YP AREA was informed by the field of positive youth development and the Typology of Youth Participation and Empowerment (TYPE) framework [30]. Positive youth development is a strengths‐based approach that views AYAs as valuable resources to be cultivated rather than risks to be managed [42]. For positive youth development to occur, AYAs must have both internal and external assets such as commitment to learning; sense of purpose; structure and support; and relationships with caring adults [43]. YP AREA promotes individual and collective AYA empowerment through participation in the research process. We bring AYAs with autoimmune conditions at different developmental stages together and provide support as they work with one another to complete research training and committee projects. The Project Team provides structure to the overall tasks of YP AREA, group education, and individual mentoring to facilitate connections and opportunities that will help CMs grow in their personal and career development. Through their work on committees, CMs build a sense of community and find their collective voice in the healthcare system. Given the CMs' range of interests and experiences, CMs teach one another and act as role models in areas where they feel more confident. Our long‐term goal for YP AREA is for AYAs with autoimmune conditions to gain the skills and confidence necessary to make lasting, meaningful changes in the healthcare system that will ultimately improve the health outcomes of other AYAs. This paper outlines the process of co‐creating this group with the inaugural cohort of CMs.

## 2 Methods

The aim of this project was to determine the feasibility of a co‐created model to train young patients to be research partners, and to evaluate their engagement in the process and potential outcomes for future iterations and study.

Project Team members [KC, JZ, CKW] convened a council of young people (16–22 years old) with autoimmune diseases who were interested in collaborating with researchers on studies. We chose to focus this project on autoimmune conditions based on the Project Team's prior research and advocacy work and their own diagnoses with autoimmune conditions. Limiting selection to those with autoimmune conditions allowed for some variation between different conditions and condition groups (rheumatic, gastrointestinal, metabolic, etc.) while also ensuring some commonalities and similar challenges between all members. These young people, called Council Members (CMs), were recruited through social media and referrals from researchers, healthcare providers, and patient organisations. The Project Team decided that 12 CMs was an ideal member for this first cohort and therefore aimed to recruit 14 AYAs in anticipation of attrition. The number of CMs was purposefully capped to ensure each member retained an opportunity to contribute to the project. CMs were recruited to reflect a range of research experiences; some were already involved as patient partners, hired as undergraduate research assistants, or conducting research for their coursework. This variety was intended to promote a reciprocal learning process, wherein CMs model leadership, exchange experiences, and create new ideas, rather than a unidirectional education process led solely by the Project Team. The aim was for at least four AYAs (approximately 1/3 of the cohort) to have no exposure to any type of research. All CMs were paid $100 for working 2–5 h per month and an additional $50 if they served on a committee. Additionally, CMs were provided an additional stipend for attending the initial retreat and if they gave a presentation (e.g., at academic meetings, patient conferences) on behalf of YP AREA.

**Figure 1:**
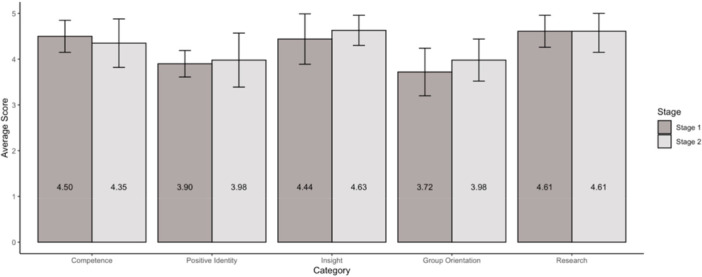
Average RES scores by survey category, Stage 1 versus Stage 2. The number of survey respondents that allowed external data sharing is 5 for the Stage 1 and 10 for Stage 2. Average values are depicted, with standard deviation visualised as error bars.

Throughout the project, we referenced the TYPE Framework to reflect on how YP AREA and all activities that occurred after recruitment would be co‐led by CMs and the Project Team. The TYPE Framework offers five types of youth participation for shared control between those in power (e.g., researchers, policy makers, healthcare providers, etc.) and AYAs [30]; (Figure 1). When the initial project funding was proposed and CMs were recruited, the Project Team made almost all decisions [Vessel/Symbolic Empowerment]. In this stage, the Project Team, along with the first two CMs, drafted the initial structure of Stages One and Two. Later, as CMs learned about research processes and gained experience with advocacy, CMs took the lead on activities and most decisions transitioned to shared control [Pluralistic/Independent Empowerment]. Several feedback mechanisms during council‐wide meetings, mentor meetings, and confidential spaces for CMs were designed to elicit CMs' critiques, preferences, and new ideas around education and empowerment activities. For example, CMs completed a survey at the end of each council‐wide meeting asking if each meeting improved research and advocacy skills paired with an open‐ended question for additional comments. CMs' responses were intended to be acted upon immediately—from incorporating new training activities and adjusting the timeline, adding another group norm to promote CMs' safety and confidentiality, to refining minor details about guest speakers and topics. At this point, the training process was less about following the Project Team's prescriptive structure, and more focused on a collaboration between the Project Team and CMs about how CMs could gain the skills needed, and be exposed to the appropriate networks and environments, to advocate for within health research. At the end of the project, CMs completed a survey about their perceived participation in meetings and committees. CMs developed, executed, participated in, and analysed the surveys and interviews, as well as led the development of this manuscript.

The process to train CMs to be patient partners in research was split into two stages. Notably, it was not our goal to train CMs to be researchers themselves, but for them to understand how research is conducted (i.e., stages of research, basic concepts of analysis, traditional methods of dissemination) and to process their lived experiences as unique expertise that allows them to meaningfully contribute to research discussions. Detailed description of activities performed, and the skills targeted are shown in Table 1.

– Before beginning the research training process, we emphasised rules of confidentiality, safety, respect, etc., and focused on building rapport and storytelling capacity within the group. We continued re‐visiting these values throughout the project.
– *Stage 1 Educate (July–December 2021)*. Stage 1 focused on exposing CMs to the various phases of the research process (from study conception to dissemination) and the value of researcher‐patient collaborations. The council held 1 weekend‐long virtual retreat focused on project onboarding, rapport and community building, and an introduction to patient advocacy in research. This was followed by 2‐h monthly virtual meetings which included didactic education, group discussions, and guest speakers. In addition, CMs completed asynchronous homework such as FYREworks Training [44], reading research articles, and attending a virtual conference. Introduced through the PCORI Engagement Tool and Resource Repository, the FYREworks Training is a structured, interactive, web‐based education portal designed for families with chronic conditions to learn about basics of research. It was chosen because of its specific tailoring to teens seeking to advise on health‐related research, as well as its self‐paced nature to allow CMs to engage as much as they need based on their previous experience with research [44]. Finally, the council formed five committees to organise projects and delegate tasks: Council Evaluation, Scientific Dissemination, Community Outreach, Grants and Roadmap, and Branding and Marketing. Participation on a committee was optional, and CMs joined committees based on their skillset (where they felt of most use) or interest (to learn or develop a new skill set). All CMs joined at least one committee, and many joined multiple.
– *Stage 2 Empower (January–December 2022)*. Transitioning to 90‐min bi‐monthly meetings, CMs reflected on their illness experiences and crafting their story to promote change in research, policy, and the AYA patient community. As a group, the council completed projects to (1) express to healthcare providers and researchers the value of AYAs as partners in research and (2) communicate to their AYA peers the importance of engaging with research to improve personal and community health. We provided space for each CM to present their story in a meaningful way and discuss aspects of their experience that were meaningful to them. Building on this storytelling experience, each CM crafted a product pertaining to their story to achieve a personal or career goal (e.g., personal statement for medical school application, patient partner biosketch, advocacy presentation).

**Table 1:** Wraparound professional development: Activities performed and corresponding goals during Stage 1 and Stage 2.

Learning objectives (CMs will…)	Activity
– Develop a basic understanding of how research is conducted – Explain the value of research in health‐related decision making	– Didactic education (e.g., lecture about the scientific method) – FYREworks training [44] – Read research article independently and present main takeaways to peers – Attend research conferences – Guest discussions with researchers about various topics and study design – Read the funding application for current award – Contribute ideas and/or write passages for new funding applications – Apply to present at research conferences – Develop this peer‐reviewed manuscript
– Identify the importance of young patient input throughout the research process	– Discussions about current gaps in patient‐facing dissemination – Guest discussions with patient advocates – Create a guide, for AYA peers by AYA CMs, about how and why to engage with research
– Advocate for good practices when including patients on the research team (skills and confidence)	– Create a guide for researchers about how to engage AYAs as patient partners in research – Present to researchers and healthcare providers at local organisations and national conferences about YP AREA
– Tell their story through a variety of modalities (skills and confidence)	– Storytelling activities (Overarching story vs in reaction to specific topics/products) – Do a Photovoice project, discuss captions, tell personal stories about how they felt about the COVID 19 pandemic – Internal presentations to learn about each other's conditions and develop rapport – Practice producing materials that share (disseminate) specific study findings with AYAs
– Develop professional skills necessary to remain updated and a productive member of a study team	– Develop and follow rules for collaborating as a safe, respectful team; Problem solve interpersonal dynamics – Maintain timely communication, assignment completion, and attendance via online platforms

Abbreviations: AYAs, adolescents and young adults; CMs, Council Members; FYRE, Family, Youth and Researcher Education; YP AREA, Young Patients' Autoimmune Research and Empowerment Alliance.

Throughout the project, AYA CMs each met with a Project Team mentor every 4–6 weeks to check in about their engagement and to establish personal goals related to research and advocacy. Individual mentoring sessions allowed the Project Team mentor to identify strengths of each CM and facilitate connections and opportunities that will help CMs grow in their personal and career development. This combination of activities was selected for a variety of reasons, including: the Project Team's experience teaching and facilitating groups, to appeal to a variety of learning styles, in response to CMs' preferences, and to expose CMs to real‐world research and make a meaningful impact as soon as possible.

We conducted an evaluation to determine the feasibility of YP AREA activities, to understand if they improved CMs' research readiness and empowerment, and to identify areas for improvement when sustaining this work. In addition, we administered one six‐question survey about the FYREworks training to evaluate how helpful the training was to increase research knowledge. Project methods were approved with Exempt status by the University of Wisconsin‐River Falls Institutional Review Board.

### 2.1 Qualitative Data

After each project stage, all CMs and Project Team members reflected on their engagement, successes, and lessons learned using an interview guide adapted from periodic reflections [40] (Appendix A). CMs participated in a 30‐min one to one interview, and Project Team members participated in a 1‐h group interview. Interviews were facilitated by a trained CM [ARK, KA]. Interviews were recorded and transcribed. Interview summaries, a step of rapid analysis [45], were created and reviewed by at least two CMs [ARK, KA] and discussed with the Project Team. The data was plotted into a table to determine CMs' and Project Team members' perspectives about the council's successes and processes to improve in the future. Summary points were quantified to determine which ideas were stated most frequently.

### 2.2 Quantitative Data

To supplement qualitative data and further understand how they responded to training activities, CMs completed the following quantitative surveys after each stage:

– Research and Empowerment Survey (RES; completed at CMs' onboarding and after Stage 1 and 2): 18 questions were extracted from the validated Service User Psychological Empowerment Scale (SUPES [46]) to measure four dimensions of psychological empowerment: competence, positive identity, insight, and group orientation. After the team was unable to find an existing standardised tool, six custom questions were developed to gauge CMs' familiarity with specific research concepts (labelled *research readiness*). CMs were asked to rate their level of confidence with (i) knowledge of research concepts, (ii) knowledge of research ethics, (iii) research skills, (iv) ability to communicate with researchers, (v) ability to communicate with peers about research, and (vi) understanding of how a patient can contribute to research.
– Patient Engagement in Research Scale (PEIRS; completed after Stage 1 and Stage 2 [47]): Developed to quantify members' meaningful engagement in research, the assessment comprised of 22 questions across seven categories, such as opportunities to contribute, experiencing positive impacts on one's life, integration into the Project Team, communication, and assisting with decision‐making.

CMs completed a consent form where they were given two options: consent for using their data internally to improve the council and/or consent for their deidentified data to be shared externally in presentations and manuscripts. Each assessment employed a 5‐point Likert scale (5 ‐ strongly agree, 1 ‐ strongly disagree) and were completed through Qualtrics. Statistical analysis and table and figure creation were conducted using R (packages used: tidyverse, reshape, ggplot2, gtsummary).

## 3 Results

### 3.1 Engagement in YP AREA Activities

Out of 45 applicants, 13 CMs were selected as the initial cohort. Two male CMs left the project within the project timeframe: one within the first month to pursue a new full‐time job opportunity. Because of timing, he was quickly replaced with another young person who applied and been interviewed. The second CM left halfway through the project because he felt the council activities were not a good fit for his interests. This resulted in a total of 12 CMs who completed the entire project period. CMs represented a variety of autoimmune conditions and geographic regions across the United States, and most identified as female (Table 2).

**Table 2:** Council members demographic summary (*N* = 12).

Demographic feature	*n* (%)
Condition	
Crohn's disease	1 (8%)
Juvenile arthritis	2 (17%)
Lupus	3 (25%)
Type 1 diabetes	3 (25%)
Multiple conditions	3 (25%)
Age at recruitment	
16–19	4 (33%)
20–22	8 (67%)
Region^a^	
East Coast	1 (8%)
Midwestern US	4 (33%)
Southern US	4 (33%)
West Coast	3 (25%)
Gender	
Female	9 (75%)
Male	2 (17%)
Non‐Binary	1 (8%)
Race^b^	
Asian	1 (8%)
Black or African American	2 (17%)
White	8 (67%)
Multiracial or Multiethnic	1 (8%)
Ethnicity	
Hispanic, Latinx, or Chicanx	3 (25%)

^a^ Many Council Members were transient, living in different states for the academic year versus breaks and holidays. These numbers represent the home with which the Council Members most resonated at the time of writing.

^b^ Race was obtained via open‐ended question.

Attendance at meetings averaged 9.0/12 members, with weekday meetings averaging 8.6 members in attendance and weekends 9.2 members. Mean scores on the PEIRS remained high across all categories at both time points (Stage 1 range: 4.6–4.8/5; Stage 2 range: 4.5–4.8/5) with limited variability, indicating CMs maintained a consistent level of perceived meaningful engagement in research through both stages of the project. When stratifying CMs by self‐reported time in council activities collected after Stage 2, CMs who reported spending four or more hours per month doing committee work had higher scores on all PEIRS categories (range of mean scores across categories: 4.7–4.9/5) than those whose participation outside of all‐council meetings was 0–4 h per month (range: 4.3–4.7/5).

### 3.2 Research Readiness

In interviews, CMs responded favourably to the educational activities in Stage 1. Due to time constraints and competing priorities, CMs occasionally found it difficult to complete asynchronous tasks that were assigned to increase their awareness of the existing landscape of research. Still, they reported learning from these assignments, particularly the FYREworks Training. CMs also emphasised that having guest speakers (e.g. researchers, experienced patient partners, community engagement specialists) were one of their favourite activities in their training. Guest speakers were a chance for CMs to obtain insight into the intricacies of research (i.e., ‘what they're doing and how they think’) and learn how to interact with professionals in this field. CMs noted that ‘[Researchers are] way more accessible than you think’ and that they are ‘people too. Talking to them wasn't that difficult’. Some CMs reported that guest speakers further empowered and encouraged them to advocate for change using their own experiences.

After Stage 1 activities, CMs understood why collaborations between researchers and patients are important. One CM stated: ‘I want to make sure that we're doing research on stuff that's important to us [patients] and I think that's really important and not always something that researchers think of’. While some still grappled with what research collaboration would look like in practice (‘I don't know enough in detail about each step of the research process where I can be like, this is the linchpin’), all CMs felt ready to act as patient partners given the opportunity. The number of research activities in which each CM collaborated because of YP AREA activities varied. Examples of CMs' patient engagement, divided by degree of involvement [48], are provided in Table 3. The Project Team discussed how CMs were creative and ambitious in their goals and vision for YP AREA and young patients in research, which led to many more research‐focused group activities (e.g., literature review, review of dissemination models, leading of research in collaboration with other groups) than originally planned and budgeted.

**Table 3:** Examples of Council Members' engagement in research.

**Inform** (‘Study plans are communicated to the patient community’)^a^	– To increase their knowledge on newest research, Project Team and CMs invited study teams to share their research within a YP AREA meeting. In turn, CMs developed age‐appropriate content that disseminated this information to their peers on social media.
**Consult** (‘Offer opinions, advice, and feedback’)	– One researcher, who was adapting a work‐related behavioural intervention, attended a YP AREA meeting to ask CMs for advice on how to be mindful of their transitional stage and/or people new to employment. – Individual CMs have served as invited keynote speakers at conferences interested in their patient experiences.
**Collaborate** (‘Joint decisions solicited; taking actions jointly’)	– Individual CMs have been added as key Project Team members on research funding applications in the areas of health equity, reproductive health, and behavioural medicine. – In conjunction with a research centre, one YP AREA committee developed research questions and surveyed over 600 people about their knowledge of research processes.
**Stakeholder Directed** (‘Leading to community‐generated research’)	– Three CMs and CW evaluated the effect of Arthritis Camp on self‐efficacy and disease self‐management in youth with Juvenile Arthritis. – Second YP AREA funding proposal was developed based on CMs' ideas and conversations about disseminating research using platforms tailored to AYAs and underserved communities. Two CMs independently wrote individual sections of the application.

Abbreviations: AYAs, adolescents and young adults; CMs, Council Members; YP AREA, Young Patients' Autoimmune Research and Empowerment Alliance.

^a^ Headings are levels of patient engagement originally used in references from the Patient‐Centred Outcomes Research Institute and the Institute for Patient‐ and Family‐Centred Care.

In surveys, CMs reported that because of meetings in Stage 1, their knowledge about advocacy and research increased (mean of 4.9, 4.8/5 respectively) and in Stage 2, they felt more empowered to partner with researchers and advocate for their peers (mean of 4.8, 4.7/5 respectively). Congruently, RES scores indicated high scores on research readiness after Stages 1 and 2 (Figure 1). By the end of Stage 2, CMs were divided in what typology that they believed YP AREA to be according to the TYPE framework, with 5 reporting independent (41.7%), 5 reporting pluralistic (41.7%), and 1 reporting symbolic (8.3%) [30]. The Project Team also noted that as CMs took on more responsibilities and decision‐making power, they were able to navigate real life challenges they may also encounter working on research teams.

### 3.3 Personal Growth

Qualitatively, almost all CMs reported a boost in confidence, though they varied widely on how they described the resulting impact. Two CMs described their growth in acceptance of their health and identity as disabled individuals: ‘It's helped me come to terms with that label, but also recognising that there's not one form of disability’. Other CMs focused on skills that applied to professional settings, such as communicating (“I'm much more able to say ‘here's what I actually need’”), being a “team player”, and leadership. Some CMs found that their work within the council resulted in changes in their career aspirations: ‘[the council gave me] a very strong sense of what I want to contribute to this community [YP AREA], the broader patient community, and to healthcare’. YP AREA provided a chance to pursue these outlets: ‘I've been able to get my foot in the door of the professional connection and legitimacy that I didn't know how to get before because I didn't have that kind of networking opportunity’.

RES scores remained stable with categories from three RES dimensions slightly increasing in average scores (positive identity, insight, group orientation) (Figure 1). The majority of CMs indicated that the council was “very helpful” (highest level on a Likert scale) to their professional development in the areas of leadership (75%), networking (67%), patient advocacy (58%), research (83%), and career path (75%), and that individual mentorship was very helpful (75%).

### 3.4 Finding Community

While the original plan for developing the council incorporated activities to build rapport between members, CMs emphasised the importance of community in a way that was unexpected to the Project Team. Half of the CMs mentioned feeling a sense of community within the council, noting like‐minded individuals and becoming friends. One CM stated: ‘The support that I feel from this council is immense’ while another mentioned, ‘I didn't really know anyone with autoimmune diseases growing up. Having a community of people that understands and are facing similar issues has been really nice’. CMs reported that they enjoyed getting to know each other and learning about other conditions and interests through sharing and workshopping their stories. CMs used time before and after meetings or asynchronous communication (text, Slack, social media, etc.) to keep in touch and encourage each other as they entered new chapters of their lives, such as graduate school or beginning their careers. CMs also seized the opportunity to learn from their peers' lived experiences when facing challenges with their health or circumstances related to having an autoimmune condition; for example, they asked each other about experiences with different treatments, advocacy in the doctor's office, workplace accommodations, and research. This sense of community could be seen influencing how some CMs viewed the future of the council and for how research should be conducted: ‘I think that everybody has their own unique perspective and their own lived experiences. And they all have to come together for us to actually do the research, put on presentations and reach out to people, and build this Council…Each of us doesn't necessarily have that same set of skills ‐ just acknowledging it's not a one‐person job, it's all of us’. The Project Team noted that fostering this sense of community likely helped with retention within the council across the 2‐year timeframe, and motivation to advocate for young patients in research despite facing the barriers of it not yet being mainstream.

### 3.5 Areas of Improvement

CMs and the Project Team identified several ways to improve membership, training activities, internal communication, and external reach of YP AREA, which are delineated in Table 4.

**Table 4:** Areas of improvement and solutions.

Topic	Area of improvement	Solution(s)
Stage 1 interviews		
Meetings	– CMs requested more frequent meetings	– Implementation of bi‐monthly meetings
	– Guest speaker diversity (CMs desired exposure to researchers conducting different types of projects)	– Guest speakers discussed a variety of research topics (e.g., self‐management, provider education) and methods (e.g., intervention development, implementation, descriptive surveys)
Activities	– More small group and asynchronous activities – Optional activities to gain skills based on varied interests	– Discussions and homework review conducted in breakout rooms as small groups – Opportunities made available to participate in optional activities (YP AREA‐led research projects, scientific presentations, advocacy invitations from patient organisations, etc.)
	– More social time to connect as a group	– 30‐min optional social time available before weekend meetings
Involvement/ responsibilities	– Difficulty keeping track of tasks and staying involved with projects	– Committee updates provided at general meetings – Transition to Slack for communication instead of email
Stage 2 interviews		
Hearing all voices	– Ensuring that all members are being heard throughout council meetings	– Use of breakout rooms, calling on others during meetings, and continued discussion through asynchronous messaging – CMs also mentioned the phrase “Step‐up and step‐back” as a reminder to CMs who are quieter to participate in the conversation and outspoken CMs to allow time for other members to speak
Recruitment	– Recruiting a new cohort for the next project – Lack of diversity among CMs in specific areas. (e.g., gender, medical condition, education)	– Coordinate targeted recruitment strategy considering gaps in current CM demographics – Recruitment through providers may help connect patients unfamiliar with research to YP AREA

Abbreviations: CMs, Council Members; YP AREA, Young Patients' Autoimmune Research and Empowerment Alliance.

## 4 Discussion

This council was created to prepare AYA patients living with chronic autoimmune disease, who often experience difficulties in transitional care and participating in research, for an active role in health research. Involvement in research helps redistribute power and give patients meaningful opportunities to influence the healthcare landscape; both may offer hope during the challenging transition into adulthood. In the presented data, we evaluated our process for training AYAs to be partners in research and empowering them to become advocates for themselves and their peers. YP AREA CMs learned how to engage in all aspects of the research process. Training activities focused on how to make meaning of their lived experiences and communicate their insights to impact research. In telling their stories and working with one another toward a common goal, CMs also formed a community of patients with shared values and experiences.

From the start, the Project Team was emphatic about its commitment to participatory values—that the group and its methods be ‘for AYAs, by AYAs’ to ensure all content remain meaningful and expectations achievable to CMs. As expected, this model had its challenges. One potential difficulty was being able to meet CMs where they were, be it their schedule, skills, or other stressors. In anticipation, the Project Team included CMs in the creation of the governance document which outlined job expectations for CMs along with policies and procedures of the council. Attendance was flexible to accommodate work schedules and the unpredictability that comes with a chronic condition if there was timely and consistent communication between each CM and their Project Team mentor. It is noteworthy that even with this accommodation, the council achieved strong attendance at meetings and scored as highly engaged in the project. In line with other research [49], one reason may be because of the community they had built—the feeling of shared experience and like‐mindedness serving as mechanisms to build capacity and confidence, thereby sustaining CMs' commitment to the project for a nearly 2 year period.

Completing this evaluation allowed us to assess our ability to overcome power dynamics and outdated methods by which researchers and young people attempt to work together: in this project, CMs initiated and executed research‐related activities that were above and beyond the initial plans of the Project Team. Specifically, CMs showed tremendous leadership and professionalism in developing and delivering scientific presentations, sharing their stories in a succinct and impactful manner with researchers and their peers, conducting the project evaluation, and writing this manuscript. Additionally, CMs applied for funding to continue the council's work, including finding the funding agency and mechanism, writing the application, and completing an interview. The TYPE framework [30] was essential in guiding the balance of who held the power and leadership roles between the council and Project Team. CMs appreciated the Project Team taking the lead in Stage 1 as they learned about partnering with researchers but were able to build their own skills as they worked in smaller committees on tasks such as YP AREA's branding and marketing, outreach to AYA peers through social media and work with other organisations, and academic dissemination of our work. Allowing CMs to take the lead on these projects helped them further develop their research‐related skills, but also their leadership and cooperation with their peers. As knowledge, competence, and confidence increased, CMs took ownership of tasks and committees, and by the end of the 2‐year project, achieved shared ownership of the entity of YP AREA. To do this work, the Project Team aimed to provide CMs with safe spaces and scaffolding. The Project Team accommodated CMs' needs (instead of the other way around), which meant getting to know them as people, honouring their voices, providing individual mentoring, and stepping back to allow CMs to practice their skills. This intentional time and structure allowed CMs to build their own sense of community and find their collective voice in the healthcare system and research.

For areas of improvement, both CMs and the Project Team noted a need to increase the diversity of CMs. While there was strong representation in some areas, there were notable gaps of applicants who were younger, identified as males, had certain autoimmune disease diagnoses (e.g., multiple sclerosis), and identify as part of populations less represented in healthcare. Most CMs also joined the project with already high levels of empowerment and were highly engaged from the start of the project, which suggests homogeneity in the knowledge and skills of current CMs. As our goal is to produce trained research partners, it is important that YP AREA and its methods be accessible to patients who can contribute a variety of patient experiences to research projects. Additionally, the YP AREA training curriculum lacked discussions on patient partners' ability to reflect on the limitations of their own experience and their role in holding research teams accountable to the concepts of health equity. Future iterations of training will be revised to include considerations from literature that challenges over‐simplified versions of patient engagement [50].

This project had several limitations. Because this was a feasibility study, data collection was intended to be exploratory of CMs' engagement and perceptions of empowerment. Therefore, it did not include an exhaustive set of pre‐post outcomes (e.g., objective measures of research knowledge or research competence; formal documentation of research experience). Additionally, only five CMs provided consent during the first time collecting RES data, likely due to the internal nature of this evaluation leading to confusion around the consent form. Finally, we encountered challenges in our attempts to measure changes in research engagement and empowerment. When crafting our evaluation to track the needs and abilities of our patient partners, we discovered a paucity of assessment tools that we were able to use for this process [51]. Consideration must be given to what is essential to being a patient partner—for example, whether it is necessary to know the fundamentals of randomised control trials if an AYA is consulting on a qualitative project. The six added items developed by the Project Team showed high scores from beginning to end, indicating a need for validated questions that are more responsive to change. As we recruit new CMs, we anticipate collecting more mixed methods data, such as a knowledge test and more robust tracking of each training component, to assess the efficacy and provide additional context around our chosen research education tools.

## 5 Conclusion

This paper describes the creation of a council of AYAs with autoimmune conditions who are dedicated to making healthcare research inclusive and accessible to young people. Our process to co‐create research training, for young patients by young patients, addresses challenges with engagement and collaboration faced by adult researchers working with this population. It also builds confidence and capacity for the AYAs who are actively seeking to be involved in this field. At the same time, co‐creation requires time, patience, and intentional reflection on power dynamics, relationship building, and flexibility to accommodate AYAs' developmental needs and busy schedules. Evaluation results of the inaugural cohort of YP AREA show that young people can achieve readiness to participate in the research process with appropriate training and support, and that leadership opportunities can empower patients to advocate for themselves and their peers. Prior to fully scaling out this programme, immediate next steps include recruiting AYAs from underrepresented demographics and co‐adapting the training to further address issues of safety, tokenism, power, and trauma. Simultaneously, we intend to work with other stakeholders, such as researchers, community organisations, and funders, to better prepare research teams to work with the AYAs who we train.

## Author Contributions

**Anya R. Khurana:** investigation, writing ‐ original draft, methodology, writing ‐ review and editing, formal analysis, data curation. **Kaye Anderson:** investigation, writing – original draft, methodology, writing – review and editing, formal analysis, data curation. **Kristine Carandang:** conceptualisation, investigation, funding acquisition, methodology, writing – review and editing, formal analysis, project administration, data curation, supervision. **Ela Chintagunta:** investigation, writing – review and editing, methodology, formal analysis, data curation. **Jennifer Ziegler:** conceptualisation, writing – review and editing, methodology, supervision, project administration, funding acquisition.

## Ethics Statement

Project methods were approved with Exempt status by the University of Wisconsin‐River Falls Institutional Review Board.

## Conflicts of Interest

The authors declare no conflicts of interest.

## Supporting information

AppendixOnly.

## Data Availability

The data that support the findings of this study are available on request from the corresponding author. The data are not publicly available due to privacy or ethical restrictions.
