# Supplementary material for: Training Adolescents and Young Adults to be Partners in Research: Co‐Creating the Young Patients' Autoimmune Research and Empowerment Alliance
Source: Health Expect. 2026 Apr 5;29(2):e70642. doi: 10.1111/hex.70642 (PMC13051984; doi:10.1111/hex.70642)
Supplement: Supplementary file 1 — AppendixOnly. [file HEX-29-e70642-s001.docx]

# Appendix A. Guide for Interviewing Council Members

**Introduction**

These reflections are intended to provide an opportunity to check in regularly about how the project is going. Our main goal is to take a few minutes to discuss, document, and reflect on key activities, events, and changes occurring over the course of implementation of the project, and in this case, Stage One/Two which was to educate and train the council/empower the council with skills to advocate for themselves and their community.

*Note to interviewer: *Guide provides suggested wording only. While as many topics should be covered in given timeframe, conversations may be different based on your style/approach.*

**Stage One Interview Questions & Prompts**

- In front of you are a list of activities that Council Members did in Stage One, you may have participated in some of these and not others. Let's talk through how you think each of these activities went.
- Do you feel educated and trained on how to work with researchers? What was most effective in training you to work with researchers?
- Are you ready to work with researchers? Why or why not?
- How would you like to work with researchers? Meeting on their level or our level? How do you envision an ideal partnership with researchers?
- Did you do the homework? Why or why not? How can we improve this in the future?
- We are having a hard time getting people to fill out the evaluation surveys, any thoughts?
- Did you feel engaged in the Stage One activities? Why or why not?
- One of the overall project goals is to slowly transition power from the Project Team to the Council Members. How do you think this is going? What can be improved about engaging the Council Members? What was the most successful example of how this happened?"
- What do you see as the biggest area of improvement from Stage One?
- Do you have any concerns or have there been barriers to your involvement in the council?
- Are there any training activities that were missing from this stage?
- What are lessons learned from Stage One?
- How could we improve the education and training of new council Members?
- What do you see as the biggest success of Stage One?
- What are the next steps going forward?
- Are there any other topics you were expecting or wanted to discuss today that I didn’t ask about?

**Stage Two Interview Questions & Prompts**

- Which committees and projects have you worked on as part of the council? What did you do in the project/committee?
- How could we recruit members to ensure inclusion of underrepresented demographics and perspectives? Is there a group that you noticed that is underrepresented in our council? How could we reach that group?
- How can we improve the format of our discussions to ensure all YP AREA members’ voices are adequately represented/heard?
- What do you see as our biggest success?
- What do you see as our biggest area of improvement for the future?
- Do you feel educated and trained on how to work with researchers? What activities were most successful in helping you learn about and get involved with research? How do you see yourself working on research or with researchers? Do you feel like you’ve grown since we started the project?
- Do you have any concerns or have there been barriers to your involvement in the council?
- Have you been able to manage the YP AREA workload? Is there something the council or Project Team could do to help with this? What prompted you to take on new tasks/projects?
- How has this project been helpful?
- Are there any other topics you were expecting or wanted to discuss today that I didn’t ask about?
